# 4,5-Diphenyl-2-*p*-tolyl-1*H*-imidazol-3-ium nitrate

**DOI:** 10.1107/S1600536809018789

**Published:** 2009-05-23

**Authors:** Li-Jing Cui

**Affiliations:** a Ordered Matter Science Research Center, College of Chemistry and Chemical Engineering, Southeast University, Nanjing 210096, People’s Republic of China

## Abstract

In the cation of the title compound, C_22_H_19_N_2_^+^·NO_3_^−^, the N atom in the 3-position of the imidazole is protonated. The three pendant aromatic rings are twisted from the plane of the imadazolium ring by dihedral angles of 38.1 (1), 43.74 (9) and 20.4 (1)°. In the crystal structure, N—H⋯O and N—H⋯(O,O) hydrogen bonds link the mol­ecules to form an infinite one-dimensional chain parallel to the *c* axis.

## Related literature

For uses of imidazole derivatives, see: Dai & Fu (2008 ▶); Fu & Xiong (2008 ▶); Huang *et al.* (2008 ▶).
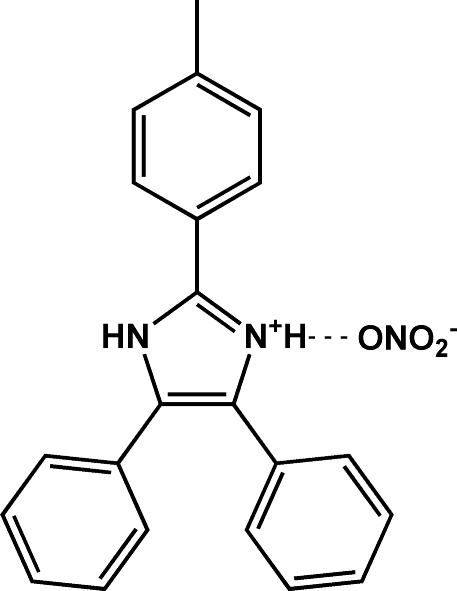


## Experimental

### 

#### Crystal data

C_22_H_19_N_2_^+^·NO_3_^−^
*M* *_r_* = 373.40
Orthorhombic, 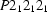

*a* = 8.442 (2) Å
*b* = 12.970 (3) Å
*c* = 17.098 (3) Å
*V* = 1872.3 (6) Å^3^
*Z* = 4
Mo *K*α radiation
μ = 0.09 mm^−1^
*T* = 298 K
0.40 × 0.35 × 0.25 mm

#### Data collection

Rigaku Mercury2 diffractometer
Absorption correction: multi-scan (*CrystalClear*; Rigaku, 2005 ▶) *T*_min_ = 0.955, *T*_max_ = 1.000 (expected range = 0.934–0.978)
19745 measured reflections
4278 independent reflections
2714 reflections with *I* > 2σ(*I*)
*R*_int_ = 0.089

#### Refinement

*R*[*F* ^2^ > 2σ(*F* ^2^)] = 0.059
*wR*(*F* ^2^) = 0.143
*S* = 1.03
4278 reflections
254 parameters
H-atom parameters constrained
Δρ_max_ = 0.26 e Å^−3^
Δρ_min_ = −0.20 e Å^−3^

### 

Data collection: *CrystalClear* (Rigaku, 2005 ▶); cell refinement: *CrystalClear*; data reduction: *CrystalClear*; program(s) used to solve structure: *SHELXS97* (Sheldrick, 2008 ▶); program(s) used to refine structure: *SHELXL97* (Sheldrick, 2008 ▶); molecular graphics: *SHELXTL/PC* (Sheldrick, 2008 ▶); software used to prepare material for publication: *SHELXTL/PC*.

## Supplementary Material

Crystal structure: contains datablocks I, global. DOI: 10.1107/S1600536809018789/im2112sup1.cif

Structure factors: contains datablocks I. DOI: 10.1107/S1600536809018789/im2112Isup2.hkl

Additional supplementary materials:  crystallographic information; 3D view; checkCIF report

## Figures and Tables

**Table 1:** Hydrogen-bond geometry (Å, °)

*D*—H⋯*A*	*D*—H	H⋯*A*	*D*⋯*A*	*D*—H⋯*A*
N2—H2*A*⋯O1^i^	0.86	2.05	2.905 (4)	176
N2—H2*A*⋯O2^i^	0.86	2.39	2.922 (3)	121
N1—H1*A*⋯O3	0.86	1.96	2.720 (3)	147

Symmetry code: (i) 
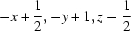
.

**Fig. 1.**
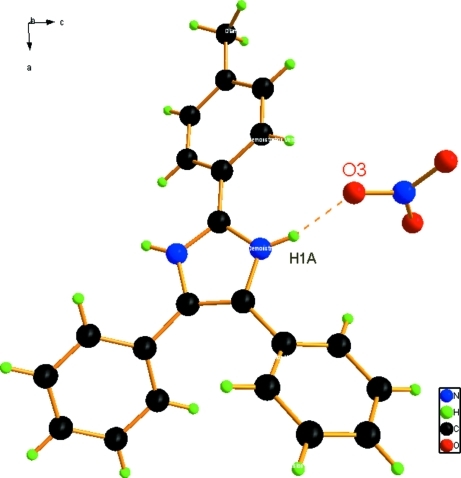
A view of the title compound with the atomic numbering scheme. Displacement ellipsoids were drawn at the 30% probability level.

**Fig. 2.**
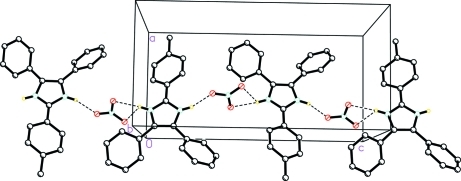
The crystal packing of the title compound viewed along the b axis showing the infinte chain realized by hydrogen bonds (dashed lines). H atoms not involved in hydrogen bonding have been omitted for clarity.

## supplementary crystallographic information

### Comment

Imidazole derivatives have found wide range of applications in coordination chemistry because of their multiple coordination modes as ligands to metal ions and for the construction of novel metal–organic frameworks (Huang *et al.*, 2008; Fu & Xiong, 2008; Dai & Fu, 2008). We report herein the crystal structure of the title compound, 4,5-diphenyl-2-*p*-tolyl-1*H*-imidazol-3-ium nitrate.

The title compound contains an organic cation and a nitrate ion in the asymmetric unit. The imidazole N atom in 3-position is protonated. Imidazole and benzene rings are twisted from each other by a dihedral angle of 38.1 (1)°, 43.74 (9)° and 20.4 (1)°. The crystal packing is stabilized by N—H···O hydrogen bonds to form an infinite one-dimensional chain parallel to *c* axis. (Table 1, Fig. 2).

### Experimental

Under nitrogen protection, 1,2-diphenyl-ethane-1,2-dione (20 mmol), 4-methylbenzaldehyde (20 mmol) and amine acetate (50 mmol) were dissolved in 60 ml of HOAc. The mixture was stirred at 110 °C for 20 h. The resulting solution was poured into ice water (200 ml) and after neutralizing the mixture with NaOH (6 mol/l) a white solid was obtained. After filtration and washing with distilled water the crude product was recrystallized from an ethanolic solution (150 ml) to which nitric acid (5 ml) was added to yield colorless block-like crystals of the title compound, suitable for X-ray analysis.

### Refinement

All H atoms attached to C atoms were fixed geometrically and treated as riding with C—H = 0.93 Å (aromatic), C—H = 0.96 Å (methyl). H atoms of the N atoms were located in difference Fourier maps and in the last stage of refinement they were treated as riding on the N atom (N—H = 0.86 Å) with *U*_iso_(H) = 1.2Ueq(N).

In the absence of significant anomalous dispersion effects, Friedel pairs were averaged.

### Crystal data

**Table:**

C_22_H_19_N_2_^+^·NO_3_^−^	*F*(000) = 784
*M**_r_* = 373.40	*D*_x_ = 1.325 Mg m^−^^3^
Orthorhombic, *P*2_1_2_1_2_1_	Mo *K*α radiation, λ = 0.71073 Å
Hall symbol: P 2ac 2ab	Cell parameters from 2714 reflections
*a* = 8.442 (2) Å	θ = 3.1–27.5°
*b* = 12.970 (3) Å	µ = 0.09 mm^−^^1^
*c* = 17.098 (3) Å	*T* = 298 K
*V* = 1872.3 (6) Å^3^	Block, colorless
*Z* = 4	0.40 × 0.35 × 0.25 mm

### Data collection

**Table:**

Rigaku Mercury2 diffractometer	4278 independent reflections
Radiation source: fine-focus sealed tube	2714 reflections with *I* > 2σ(*I*)
graphite	*R*_int_ = 0.089
Detector resolution: 13.6612 pixels mm^-1^	θ_max_ = 27.5°, θ_min_ = 3.1°
CCD profile fitting scans	*h* = −10→10
Absorption correction: multi-scan (*CrystalClear*; Rigaku, 2005)	*k* = −16→16
*T*_min_ = 0.955, *T*_max_ = 1.000	*l* = −22→22
19745 measured reflections	

### Refinement

**Table:**

Refinement on *F*^2^	Primary atom site location: structure-invariant direct methods
Least-squares matrix: full	Secondary atom site location: difference Fourier map
*R*[*F*^2^ > 2σ(*F*^2^)] = 0.059	Hydrogen site location: inferred from neighbouring sites
*wR*(*F*^2^) = 0.143	H-atom parameters constrained
*S* = 1.03	*w* = 1/[σ^2^(*F*_o_^2^) + (0.0541*P*)^2^ + 0.288*P*] where *P* = (*F*_o_^2^ + 2*F*_c_^2^)/3
4278 reflections	(Δ/σ)_max_ < 0.001
254 parameters	Δρ_max_ = 0.26 e Å^−^^3^
0 restraints	Δρ_min_ = −0.20 e Å^−^^3^

### Special details

**Table:**

Geometry. All e.s.d.'s (except the e.s.d. in the dihedral angle between two l.s. planes) are estimated using the full covariance matrix. The cell e.s.d.'s are taken into account individually in the estimation of e.s.d.'s in distances, angles and torsion angles; correlations between e.s.d.'s in cell parameters are only used when they are defined by crystal symmetry. An approximate (isotropic) treatment of cell e.s.d.'s is used for estimating e.s.d.'s involving l.s. planes.
Refinement. Refinement of *F*^2^ against ALL reflections. The weighted *R*-factor *wR* and goodness of fit *S* are based on *F*^2^, conventional *R*-factors *R* are based on *F*, with *F* set to zero for negative *F*^2^. The threshold expression of *F*^2^ > σ(*F*^2^) is used only for calculating *R*-factors(gt) *etc*. and is not relevant to the choice of reflections for refinement. *R*-factors based on *F*^2^ are statistically about twice as large as those based on *F*, and *R*-factors based on ALL data will be even larger.

### Fractional atomic coordinates and isotropic or equivalent isotropic displacement parameters (Å^2^)

**Table:**

	*x*	*y*	*z*	*U*_iso_*/*U*_eq_	
N1	0.3099 (3)	0.44418 (17)	0.66981 (13)	0.0415 (6)	
H1A	0.2804	0.4503	0.7177	0.050*	
N2	0.3114 (3)	0.45286 (18)	0.54570 (13)	0.0438 (6)	
H2A	0.2827	0.4654	0.4984	0.053*	
C16	0.0757 (3)	0.5305 (2)	0.60980 (17)	0.0409 (7)	
C8	0.4517 (3)	0.4054 (2)	0.56608 (16)	0.0402 (7)	
C19	−0.2150 (3)	0.6340 (2)	0.61119 (19)	0.0464 (7)	
C7	0.4501 (3)	0.3994 (2)	0.64587 (15)	0.0368 (6)	
C15	0.2268 (3)	0.4767 (2)	0.60831 (17)	0.0407 (7)	
C6	0.5633 (3)	0.3576 (2)	0.70270 (15)	0.0389 (7)	
C1	0.6475 (3)	0.2682 (2)	0.68690 (17)	0.0462 (7)	
H1	0.6301	0.2329	0.6403	0.055*	
C21	0.0283 (4)	0.5898 (2)	0.54664 (17)	0.0482 (7)	
H21	0.0938	0.5955	0.5031	0.058*	
C10	0.7240 (4)	0.3933 (2)	0.51284 (18)	0.0492 (8)	
H10	0.7630	0.4213	0.5591	0.059*	
C17	−0.0230 (3)	0.5248 (2)	0.67441 (17)	0.0483 (8)	
H17	0.0068	0.4859	0.7177	0.058*	
C9	0.5648 (3)	0.3741 (2)	0.50526 (16)	0.0406 (7)	
C12	0.7709 (4)	0.3280 (3)	0.38461 (18)	0.0559 (8)	
H12	0.8403	0.3138	0.3438	0.067*	
C20	−0.1144 (4)	0.6400 (2)	0.54785 (18)	0.0499 (8)	
H20	−0.1442	0.6793	0.5048	0.060*	
C14	0.5107 (4)	0.3290 (3)	0.43701 (17)	0.0550 (8)	
H14	0.4035	0.3142	0.4315	0.066*	
C13	0.6136 (4)	0.3057 (3)	0.37721 (18)	0.0611 (9)	
H13	0.5761	0.2749	0.3318	0.073*	
C4	0.6997 (4)	0.3718 (3)	0.82531 (18)	0.0594 (9)	
H4	0.7178	0.4068	0.8719	0.071*	
C11	0.8262 (4)	0.3713 (3)	0.45231 (19)	0.0577 (9)	
H11	0.9335	0.3860	0.4575	0.069*	
C18	−0.1656 (3)	0.5769 (2)	0.67453 (17)	0.0475 (8)	
H18	−0.2300	0.5733	0.7186	0.057*	
C5	0.5878 (4)	0.4079 (2)	0.77306 (16)	0.0482 (8)	
H5	0.5287	0.4662	0.7852	0.058*	
C2	0.7562 (4)	0.2313 (3)	0.73960 (19)	0.0583 (9)	
H2	0.8112	0.1708	0.7290	0.070*	
C3	0.7842 (4)	0.2839 (3)	0.8083 (2)	0.0635 (9)	
H3	0.8603	0.2600	0.8431	0.076*	
C22	−0.3743 (4)	0.6851 (3)	0.6105 (2)	0.0662 (9)	
H22A	−0.4143	0.6869	0.5580	0.099*	
H22B	−0.3645	0.7542	0.6300	0.099*	
H22C	−0.4460	0.6470	0.6431	0.099*	
O3	0.1692 (3)	0.3887 (2)	0.80660 (12)	0.0721 (7)	
N3	0.1779 (4)	0.4295 (2)	0.87181 (16)	0.0564 (7)	
O2	0.1035 (4)	0.3954 (2)	0.92659 (14)	0.0901 (9)	
O1	0.2677 (3)	0.5050 (2)	0.88344 (16)	0.0796 (8)	

### Atomic displacement parameters (Å^2^)

**Table:**

	*U*^11^	*U*^22^	*U*^33^	*U*^12^	*U*^13^	*U*^23^
N1	0.0432 (13)	0.0476 (14)	0.0338 (12)	0.0053 (12)	0.0035 (11)	−0.0016 (10)
N2	0.0445 (15)	0.0562 (15)	0.0306 (12)	0.0085 (12)	0.0007 (11)	0.0029 (11)
C16	0.0384 (15)	0.0424 (15)	0.0419 (16)	0.0045 (13)	−0.0010 (15)	−0.0022 (14)
C8	0.0360 (15)	0.0432 (16)	0.0413 (16)	0.0010 (14)	−0.0024 (13)	0.0032 (13)
C19	0.0426 (16)	0.0425 (15)	0.0541 (18)	0.0026 (14)	−0.0014 (16)	−0.0071 (15)
C7	0.0331 (14)	0.0398 (15)	0.0376 (15)	0.0001 (13)	0.0015 (12)	−0.0009 (12)
C15	0.0402 (15)	0.0446 (16)	0.0374 (16)	0.0030 (13)	0.0008 (14)	0.0038 (14)
C6	0.0367 (15)	0.0470 (16)	0.0330 (14)	−0.0035 (14)	0.0009 (12)	0.0024 (12)
C1	0.0410 (16)	0.0520 (17)	0.0456 (17)	0.0058 (15)	−0.0002 (14)	0.0000 (15)
C21	0.0508 (18)	0.0513 (17)	0.0426 (17)	0.0061 (16)	0.0029 (15)	0.0078 (14)
C10	0.0453 (17)	0.0503 (17)	0.0522 (18)	0.0002 (15)	−0.0004 (14)	−0.0071 (15)
C17	0.0475 (17)	0.0576 (18)	0.0396 (17)	0.0040 (15)	0.0006 (15)	0.0048 (14)
C9	0.0432 (16)	0.0400 (15)	0.0387 (15)	0.0063 (14)	0.0025 (13)	0.0037 (13)
C12	0.057 (2)	0.068 (2)	0.0424 (18)	0.0168 (17)	0.0100 (17)	−0.0034 (17)
C20	0.057 (2)	0.0465 (17)	0.0460 (18)	0.0091 (16)	−0.0023 (15)	0.0046 (15)
C14	0.0473 (19)	0.075 (2)	0.0425 (18)	0.0035 (17)	−0.0005 (15)	−0.0022 (16)
C13	0.062 (2)	0.082 (2)	0.0386 (18)	0.0152 (19)	−0.0057 (16)	−0.0114 (17)
C4	0.070 (2)	0.068 (2)	0.0398 (17)	−0.014 (2)	−0.0128 (17)	0.0063 (16)
C11	0.0453 (19)	0.062 (2)	0.066 (2)	−0.0002 (17)	0.0112 (17)	−0.0041 (18)
C18	0.0433 (16)	0.0556 (18)	0.0435 (17)	−0.0022 (15)	0.0109 (14)	−0.0055 (15)
C5	0.0542 (19)	0.0508 (18)	0.0396 (17)	−0.0052 (17)	−0.0005 (15)	0.0020 (14)
C2	0.0446 (19)	0.067 (2)	0.063 (2)	0.0116 (17)	−0.0022 (17)	0.0084 (18)
C3	0.0506 (19)	0.083 (2)	0.057 (2)	−0.0026 (19)	−0.0162 (17)	0.023 (2)
C22	0.0498 (19)	0.064 (2)	0.085 (2)	0.0097 (16)	−0.002 (2)	−0.001 (2)
O3	0.0800 (17)	0.1055 (19)	0.0309 (12)	0.0005 (15)	−0.0044 (12)	−0.0037 (13)
N3	0.0602 (18)	0.0612 (18)	0.0477 (17)	0.0134 (15)	−0.0019 (15)	0.0046 (14)
O2	0.142 (3)	0.0865 (19)	0.0423 (13)	−0.011 (2)	0.0201 (16)	0.0009 (14)
O1	0.0746 (17)	0.0722 (16)	0.092 (2)	−0.0013 (15)	0.0004 (17)	−0.0109 (15)

### Geometric parameters (Å, °)

**Table:**

N1—C15	1.333 (3)	C17—H17	0.9300
N1—C7	1.381 (3)	C9—C14	1.383 (4)
N1—H1A	0.8600	C12—C13	1.364 (5)
N2—C15	1.324 (3)	C12—C11	1.369 (4)
N2—C8	1.379 (4)	C12—H12	0.9300
N2—H2A	0.8600	C20—H20	0.9300
C16—C21	1.385 (4)	C14—C13	1.375 (4)
C16—C17	1.386 (4)	C14—H14	0.9300
C16—C15	1.454 (4)	C13—H13	0.9300
C8—C7	1.367 (4)	C4—C3	1.376 (5)
C8—C9	1.469 (4)	C4—C5	1.382 (4)
C19—C18	1.377 (4)	C4—H4	0.9300
C19—C20	1.379 (4)	C11—H11	0.9300
C19—C22	1.499 (4)	C18—H18	0.9300
C7—C6	1.467 (4)	C5—H5	0.9300
C6—C5	1.384 (4)	C2—C3	1.378 (5)
C6—C1	1.387 (4)	C2—H2	0.9300
C1—C2	1.372 (4)	C3—H3	0.9300
C1—H1	0.9300	C22—H22A	0.9600
C21—C20	1.370 (4)	C22—H22B	0.9600
C21—H21	0.9300	C22—H22C	0.9600
C10—C9	1.372 (4)	O3—N3	1.236 (3)
C10—C11	1.377 (4)	N3—O2	1.212 (3)
C10—H10	0.9300	N3—O1	1.254 (4)
C17—C18	1.380 (4)		
			
C15—N1—C7	110.5 (2)	C13—C12—C11	119.8 (3)
C15—N1—H1A	124.7	C13—C12—H12	120.1
C7—N1—H1A	124.7	C11—C12—H12	120.1
C15—N2—C8	111.3 (2)	C21—C20—C19	121.8 (3)
C15—N2—H2A	124.4	C21—C20—H20	119.1
C8—N2—H2A	124.4	C19—C20—H20	119.1
C21—C16—C17	118.5 (3)	C13—C14—C9	120.8 (3)
C21—C16—C15	120.4 (3)	C13—C14—H14	119.6
C17—C16—C15	121.1 (3)	C9—C14—H14	119.6
C7—C8—N2	105.6 (2)	C12—C13—C14	120.0 (3)
C7—C8—C9	134.2 (3)	C12—C13—H13	120.0
N2—C8—C9	120.2 (2)	C14—C13—H13	120.0
C18—C19—C20	117.5 (3)	C3—C4—C5	119.9 (3)
C18—C19—C22	121.1 (3)	C3—C4—H4	120.1
C20—C19—C22	121.4 (3)	C5—C4—H4	120.1
C8—C7—N1	106.3 (2)	C12—C11—C10	120.4 (3)
C8—C7—C6	132.6 (3)	C12—C11—H11	119.8
N1—C7—C6	121.2 (2)	C10—C11—H11	119.8
N2—C15—N1	106.3 (2)	C19—C18—C17	121.8 (3)
N2—C15—C16	126.8 (3)	C19—C18—H18	119.1
N1—C15—C16	126.9 (3)	C17—C18—H18	119.1
C5—C6—C1	119.1 (3)	C4—C5—C6	120.3 (3)
C5—C6—C7	120.0 (3)	C4—C5—H5	119.8
C1—C6—C7	120.9 (3)	C6—C5—H5	119.8
C2—C1—C6	120.4 (3)	C1—C2—C3	120.1 (3)
C2—C1—H1	119.8	C1—C2—H2	119.9
C6—C1—H1	119.8	C3—C2—H2	119.9
C20—C21—C16	120.4 (3)	C4—C3—C2	120.1 (3)
C20—C21—H21	119.8	C4—C3—H3	120.0
C16—C21—H21	119.8	C2—C3—H3	120.0
C9—C10—C11	120.3 (3)	C19—C22—H22A	109.5
C9—C10—H10	119.8	C19—C22—H22B	109.5
C11—C10—H10	119.8	H22A—C22—H22B	109.5
C18—C17—C16	120.0 (3)	C19—C22—H22C	109.5
C18—C17—H17	120.0	H22A—C22—H22C	109.5
C16—C17—H17	120.0	H22B—C22—H22C	109.5
C10—C9—C14	118.7 (3)	O2—N3—O3	120.7 (3)
C10—C9—C8	121.3 (3)	O2—N3—O1	118.4 (3)
C14—C9—C8	120.0 (3)	O3—N3—O1	120.9 (3)

### Hydrogen-bond geometry (Å, °)

**Table:**

*D*—H···*A*	*D*—H	H···*A*	*D*···*A*	*D*—H···*A*
N2—H2A···O1^i^	0.86	2.05	2.905 (4)	176
N2—H2A···O2^i^	0.86	2.39	2.922 (3)	121
N1—H1A···O3	0.86	1.96	2.720 (3)	147

Symmetry codes: (i) −*x*+1/2, −*y*+1, *z*−1/2.
